# Supplementary material for: Predictive Value of Carcinoembryonic Antigen in Symptomatic Patients without Colorectal Cancer: A Post-Hoc Analysis within the COLONPREDICT Cohort
Source: Diagnostics (Basel). 2020 Dec 2;10(12):1036. doi: 10.3390/diagnostics10121036 (PMC7770570; doi:10.3390/diagnostics10121036)
Supplement: Supplementary file 1 [file diagnostics-10-01036-s001.pdf]

**Supplementary Table 1.** Time to cancer diagnosis based on CEA level and type of cancer.

| Type of Cancer | | Total | CEA ≤ 3 ng/mL (*n* = 1193) Time to Diagnosis | | | | | | Total[2] | Risk[3] | CEA > 3 ng/mL (*n* = 238) Time to Diagnosis | | | | | | Total[5] | Risk[6] |
|---|---|---|---|---|---|---|---|---|---|---|---|---|---|---|---|---|---|---|
| | | | 0–12 months[1] | | 13–24 months[1] | | >24 months[1] | | | | 0-12 months[4] | | 13–24 months[4] | | >24 months[4] | | | |
| H&N | | 1 | 1 | 100.0 | 0 | 0.0 | 0 | 0.0 | 1 | 0.08 | 0 | NA | 0 | NA | 0 | NA | 0 | 0.00 |
| GIC | Oesophageal | 2 | 1 | 50.0 | 0 | 0.0 | 1 | 50.0 | 2 | 0.17 | 0 | NA | 0 | NA | 0 | NA | 0 | 0.00 |
| GIC | Gastric | 15 | 7 | 63.6 | 2 | 18.2 | 2 | 18.2 | 11 | 0.92 | 4 | 100.0 | 0 | 0.0 | 0 | 0.0 | 4 | 1.68 |
| GIC | Small Bowell | 3 | 1 | 50.0 | 0 | 0.0 | 1 | 50.0 | 2 | 0.17 | 1 | 100.0 | 0 | 0.0 | 0 | 0.0 | 1 | 0.42 |
| GIC | Ampullary | 2 | 0 | NA | 0 | NA | 0 | NA | 0 | 0.00 | 2 | 100.0 | 0 | 0.0 | 0 | 0.0 | 2 | 0.84 |
| GIC | CRC | 8 | 1 | 25.0 | 1 | 25.0 | 2 | 50.0 | 4 | 0.34 | 2 | 50.0 | 0 | 0.0 | 2 | 50.0 | 4 | 1.68 |
| Hepatobiliary | Gallbladder | 2 | 1 | 50.0 | 1 | 50.0 | 0 | 0.0 | 2 | 0.17 | 0 | NA | 0 | NA | 0 | NA | 0 | 0.00 |
| Hepatobiliary | Pancreas | 3 | 1 | 50.0 | 0 | 0.0 | 1 | 50.0 | 2 | 0.17 | 1 | 100.0 | 0 | 0.0 | 0 | 0.0 | 1 | 0.42 |
| Hepatobiliary | Hepatocarcinoma | 4 | 1 | 50.0 | 0 | 0.0 | 1 | 50.0 | 2 | 0.17 | 2 | 100.0 | 0 | 0.0 | 0 | 0.0 | 2 | 0.84 |
| Skin | Basal cell | 4 | 1 | 33.3 | 1 | 33.3 | 1 | 33.3 | 3 | 0.25 | 0 | 0.0 | 0 | 0.0 | 1 | 100.0 | 1 | 0.42 |
| Skin | Melanoma | 1 | 1 | 100.0 | 0 | 0.0 | 0 | 0.0 | 1 | 0.08 | 0 | NA | 0 | NA | 0 | NA | 0 | 0.00 |
| Skin | Unspecified | 7 | 3 | 50.0 | 3 | 50.0 | 0 | 0.0 | 6 | 0.50 | 0 | 0.0 | 0 | 0.0 | 1 | 100.0 | 1 | 0.42 |
| Ginaecology | Endometrial | 2 | 0 | 0.0 | 0 | 0.0 | 2 | 100.0 | 2 | 0.17 | 0 | NA | 0 | NA | 0 | NA | 0 | 0.00 |
| Ginaecology | Ovarian | 4 | 1 | 50.0 | 0 | 0.0 | 1 | 50.0 | 2 | 0.17 | 2 | 100.0 | 0 | 0.0 | 0 | 0.0 | 2 | 0.84 |
| Ginaecology | Breast | 8 | 0 | 0.0 | 2 | 40.0 | 3 | 60.0 | 5 | 0.42 | 0 | 0.0 | 2 | 66.7 | 1 | 33.3 | 3 | 1.26 |
| Respiratory | Lung | 9 | 1 | 25.0 | 0 | 0.0 | 3 | 75.0 | 4 | 0.34 | 3 | 60.0 | 2 | 40.0 | 0 | 0.0 | 5 | 2.10 |
| Urology | Kidney | 5 | 0 | 0.0 | 2 | 66.7 | 1 | 33.3 | 3 | 0.25 | 1 | 50.0 | 0 | 0.0 | 1 | 50.0 | 2 | 0.84 |
| Urology | Urotelial | 1 | 0 | 0.0 | 0 | 0.0 | 1 | 100.0 | 1 | 0.08 | 0 | NA | 0 | NA | 0 | NA | 0 | 0.00 |
| Urology | Prostate | 10 | 2 | 22.2 | 1 | 11.1 | 6 | 66.7 | 9 | 0.75 | 0 | 0.0 | 1 | 100.0 | 0 | 0.0 | 1 | 0.42 |
| Urology | Vesical | 5 | 3 | 60.0 | 0 | 0.0 | 2 | 40.0 | 5 | 0.42 | 0 | NA | 0 | NA | 0 | NA | 0 | 0.00 |
| Haematology | Leukemia | 1 | 0 | 0.0 | 0 | 0.0 | 1 | 100.0 | 1 | 0.08 | 0 | NA | 0 | NA | 0 | NA | 0 | 0.00 |
| Haematology | Lymphoma | 6 | 3 | 60.0 | 0 | 0.0 | 2 | 40.0 | 5 | 0.42 | 1 | 100.0 | 0 | 0.0 | 0 | 0.0 | 1 | 0.42 |
| Haematology | Myeloma | 3 | 1 | 33.3 | 0 | 0.0 | 2 | 66.7 | 3 | 0.25 | 0 | NA | 0 | NA | 0 | NA | 0 | 0.00 |
| Haematology | Unspecified | 2 | 0 | 0.0 | 1 | 100.0 | 0 | 0.0 | 1 | 0.08 | 1 | 100.0 | 0 | 0.0 | 0 | 0.0 | 1 | 0.42 |
| Other | Glioblastoma | 1 | 0 | 0.0 | 0 | 0.0 | 1 | 100.0 | 1 | 0.08 | 0 | NA | 0 | NA | 0 | NA | 0 | 0.00 |
| Other | Schwannoma | 1 | 1 | 100.0 | 0 | 0.0 | 0 | 0.0 | 1 | 0.08 | 0 | NA | 0 | NA | 0 | NA | 0 | 0.00 |

| | | | | | | | | | | | | | | | | | |
|---|---|---|---|---|---|---|---|---|---|---|---|---|---|---|---|---|---|
| Kaposi | 1 | 0 | 0.0 | 1 | 100.0 | 0 | 0.0 | 1 | 0.08 | 0 | NA | 0 | NA | 0 | NA | 0 | 0.00 |
| Cancer of unknown origin | 4 | 0 | 0.0 | 3 | 75.0 | 1 | 25.0 | 4 | 0.34 | 0 | NA | 0 | NA | 0 | NA | 0 | 0.00 |
| Total | 115 | 31 | 36.9 | 18 | 21.4 | 35 | 41.7 | 84 | 7.04 | 20 | 64.5 | 5 | 16.1 | 6 | 19.4 | 31 | 13.03 |

CEA = carcinoembryonic antigen; CRC = colorectal cancer; GIC = gastrointestinal cancer; H&N = head and neck cancer; NA = not applicable; 1 number of cancer diagnosed for each period of time from baseline colonoscopy and their percentage with respect to total diagnosed cancer of the same type in patients with CEA ≤ 3 ng/mL; 2 Total number of each type diagnosed cancer in patients with CEA ≤ 3 ng/mL; 3 Incidence of each type of cancer in patients with CEA ≤ 3 ng/mL; 4 number of cancer diagnosed for each period of time from baseline colonoscopy and their percentage with respect to total diagnosed cancer of the same type in patients with CEA > 3 ng/mL; 5 Total number of each type diagnosed cancer in patients with CEA > 3 ng/mL; 6 Incidence of each type of cancer in patients with CEA > 3 ng/mL;.
